
JOURNAL INFORMATION
==============================
NLM Title Abbreviation: ScientificWorldJournal
Journal ID: ScientificWorldJournal
NLM Journal ID: 101131163
Journal ID: TSWJ

The Scientific World Journal

ISSN: 2356-6140
EISSN: 1537-744X
Publisher: Wiley

ARTICLE INFORMATION
==============================
PMCID: PMC6009286
PMID: 29973788
DOI: 10.1100/tsw.2002.14
Article ID: 130474
Article version: 1
Subjects: Short Report

Pattern of Organization of Human Mitochondrial Pseudogenes in the Nuclear Chromosomes

Woischnik Markus *
Moraes Carlos T.
Department of Neurology, University of Miami–School of Medicine, 1095 NW 14 Terrace, LPLC 3-01, Miami, FL 33136, USA
*Markus Woischnik: mwoischnik@miamiproject.med.miami.edu
Publication date: 2002
Electronic publication date: 2002 Jun 12
Volume: 2
First page: 27
Last page: 30
Copyright: Copyright © 2002 Markus Woischnik and Carlos T. Moraes.
Copyright year: 2002
License: This is an open access article distributed under the Creative Commons Attribution License, which permits unrestricted use, distribution, and reproduction in any medium, provided the original work is properly cited.
License URL: https://creativecommons.org/licenses/by/3.0/

Funding: National Institutes of Health GM55766

==============================
Advances in Gene Technology: The Genome and Beyond –
Structural Biology for Medicine (Proceedings of the 2002 Miami
*Nature Biotechnology* Winter Symposium)
*TheScientificWorld* 2002, 2(S2), 27–30
ISSN 1532-2246; DOI 10.1100/tsw.2002.14

### PATTERN OF ORGANIZATION OF HUMAN MITOCHONDRIAL PSEUDOGENES IN THE NUCLEAR CHROMOSOMES

Markus Woischnik and Carlos T. Moraes
Department of Neurology, University of Miami – School of Medicine, 1095 NW 14th Terrace, LPLC 3-01, Miami, FL 33136
MWoischnik@miamiproject.med.miami.edu

**INTRODUCTION**. Mitochondrial pseudogenes in the human nuclear genome have been previously described, mostly as a source of artifacts during the analysis of the mitochondrial genome. With the availability of the complete human genome sequence, we performed a comprehensive analysis, quantitative and qualitative, of mtDNA insertions into the nuclear genome.

**METHOD**. We used the BLAST search tool[1] to screen the public[2] and the private[3] genome draft versions for the presence of mtDNA homologues. The information, which we extracted from the obtained BLAST reports, was then used to create a map of pseudogenes, and to perform further analyses.

**RESULTS**. We found 615 independent integrations of mtDNA into the nuclear genome , that are evenly distributed among all chromosomes, as well as within each individual chromosome (Fig. 1). These pseudogenes account for a content of at least 0.016% of the human nuclear DNA. Up to 30% of a chromosome's mtDNA pseudogene content is comprised of fragments that encompass two or more adjacent mitochondrial genes. Also, we found no correlation between the abundance of mitochondrial transcripts in mitochondria[4] and the multiplicity of integrations (Fig. 2), suggesting that the transfer did not occur through RNA intermediates. This view is further supported by the significant number of integrations of fragments containing two adjacent genes, that are translated from different RNA species[4]. Comparison of the public accessible genome version with the private one from CELERA Genomics revealed little variations in the number of integrations, however, we noted discrepancies in the absolute placement of mitochondrial pseudogenes on the chromosomes between these two data sets.

Figure 1 (Woischnik & Moraes)

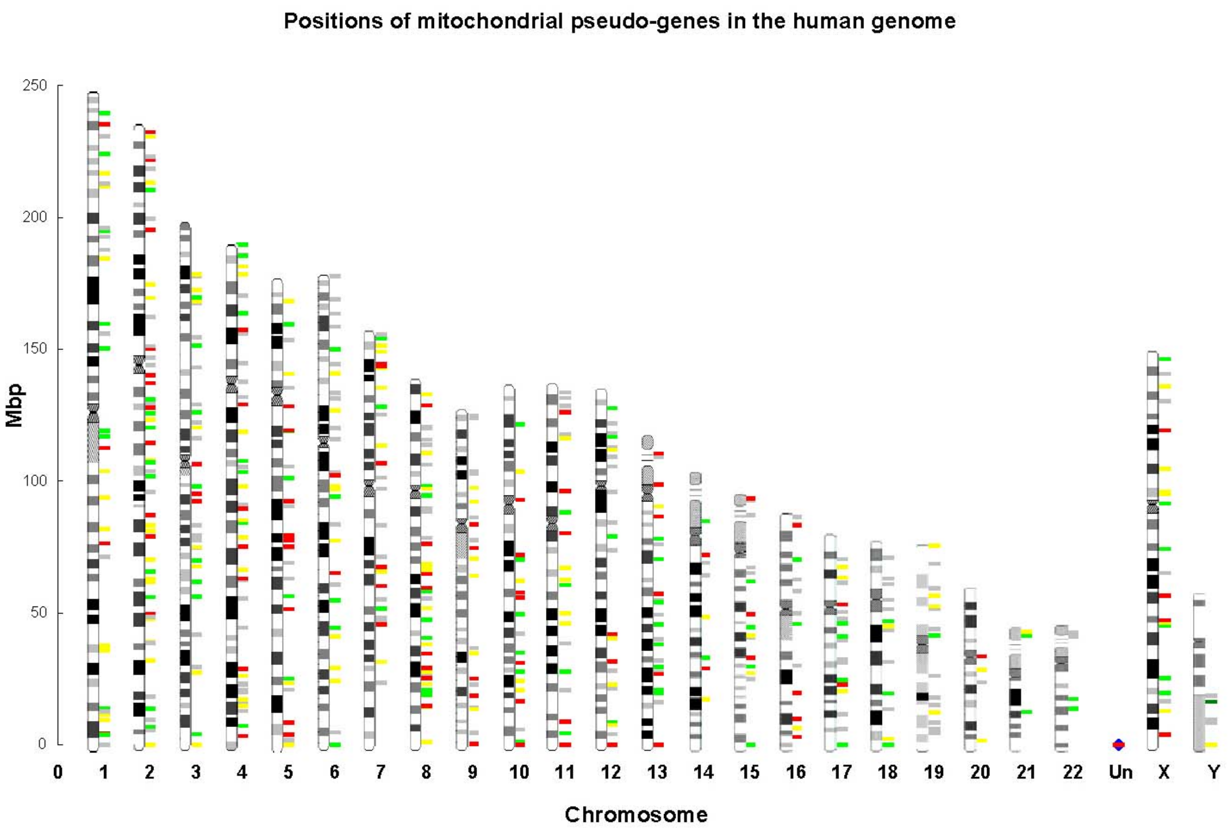

Figure 2 (Woischnik & Moraes)

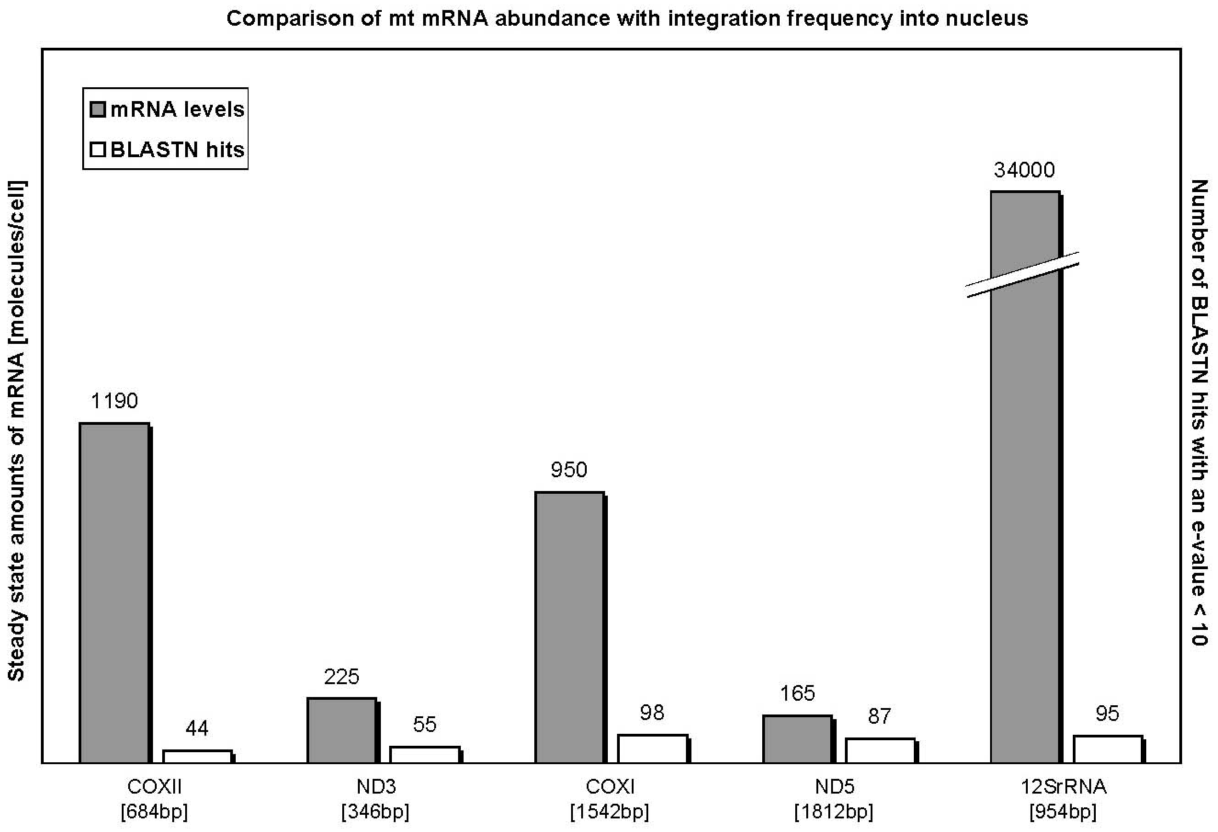

**DISCUSSION**. The extent of integration of mtDNA into the nuclear genome, both, in number and in size of the fragments, is large. The relatively high content of at least 0.016% nonfunctional mtDNA inside the genome can be explained in part by the high content of noncoding DNA on the chromosomes. Insertions of mtDNA in open reading frames would lead to interruptions in translation, because of the different codon usage for nuclear and mtDNA genes. Whenever we found an integration into a gene-coding region, it was located in an intron (data not shown). The inserted DNA sequences, as well as the frequency, and the organization of mtDNA genes on the larger fragments suggest a predominantly DNA driven transfer of mtDNA into the genome. Phylogenetic analysis suggests that mitochondrial pseudogenes are the result of a continuous transfer from the organelle to the nucleus. Because of the limited window of opportunity for mtDNA transfer to the germline, sperm mtDNA, which is released from degenerating mitochondria after fertilization, could be an important source of nuclear mtDNA pseudogenes. Both genome drafts, public and private, support our findings about the extent of mtDNA transfer. However, the discrepancies in sequence location within a given chromosome and among all chromosomes between these datasets suggest that both genome versions must be refined for the final assembly of these DNA sequences.

**ACKNOWLEDGMENT**. This work was supported by National Institute of Health Grant GM55766.

### REFERENCES

1. Altschul, S.F., Gish, W., Miller, W., Myers, E.W., and Lipman, D.J. (1990) J. Mol. Biol. 215, 403–410.
2. Lander, E.S., Linton, L.M., et al. (2001) Nature 409, 860–921.
3. Venter, J.C., Adams, M.D., et al. (2001) Science 291, 1304–1351; CELERA Genomics, Rockville, MD, http//www.celera.com.
4. Attardi, G., Chomyn, A., et al. (1990) Biochem. Soc. Trans. 18, 509–513.
